# Psychological Patient Reactions after Septorhinoplasty - Our Personal View

**DOI:** 10.3889/oamjms.2015.100

**Published:** 2015-10-30

**Authors:** Gabriela Kopacheva-Barsova, Nikola Nikolovski, Slavica Arsova, Dragoslav Kopachev

**Affiliations:** 1 *University Hospital for Ear, Nose and Throat, University Campus “St. Mother Theresa”, Skopje, Macedonia*; 2 *University Hospital of Psychiatry, University Campus “St. Mother Theresa”, Skopje, Macedonia*; 3 *Institute for Mental Health, Skopje, Macedonia*

**Keywords:** rhino/septoplasty, Patients selection for septorhinoplasty, psychological abilities, “Self-body image” questioner, Brief Symptom Inventory (BSI) test

## Abstract

**AIM:**

The aim of our study is to observe adequate and inadequate psychological reactions in patients who are candidates for septorhinoplasty, before and after surgery and to create an adequate psychological model of a person suitable for septorhinoplasty in this group of patients.

**MATERIAL AND METHODS:**

In this study, 140 patients with nasal septal deviation (deviatio septi nasi), alone or together with other nasal deformities, were observed in the period of 4 years (2011-2015 year). Our patients were psychologically observed using two standard psychological tests: Patients selection for septorhinoplasty and their psychological abilities (“Self-body image” questionnaire) and Brief Symptom Inventory (BSI) test.

**RESULTS:**

Most of the patients 43 (39.8%), thought that after rhinoseptoplasty their self-confidence arise, 32 (29.63%) expected changing’s in their life’s, few of them 9 (8, 3%) thought that the environment will act different with them. The Brief Symptom Inventory (BSI) in women group was shown that most of the women patients presented symptoms of somatisation; 23 (23.33%) and 15 (25%) one year after surgery.

**CONCLUSIONS:**

The patients made a sound decision for intervention, which was useful for the surgeon too, because it helped them choose an adequate operative technique and especially helped them in the postoperative period.

## Introduction

The desire to strive for facial beauty is not surprising that cosmetic and functional rhino/septoplasty (RSP) ranks among the most commonly performed surgical procedures all over the world. The growing popularity of RSP can be attributed to several independent factors. The most important are: better surgical techniques, safer and more comfortable anesthesia, and shorter recovering period. Reality TV shows, surgical documentaries and plastic surgery dramas have all ignited a flurry of cosmetic surgical activities, so the borders when, where and how the rhinosptoplasty will be prepared are completely dislocated. At least is not taking care about the age, sex, nationality and religion habits, socio-economic status of the patient who is searching for RSP. That leads to real problem; who, then, is the “ideal” candidate for rhino/septoplasty?

However, the surgeon instead of skill, knowledge of the operative techniques, has to faced with this big and important moment - to choose an adequate and psychological stabile candidate for RSP. That is important, because beside good postoperative results and “satisfied surgeon” we can faced with “unsatisfied” patient which inadequate psychological reactions can have an influence not only to surgeon’s work, and psychologically too.

There are two major reasons for rejecting a patient seeking RSP. One is, anatomical unsuitability; the other is emotional inadequacy [1-3]. Second, is more important. Rhinosurgeon must early learn to differentiate between healthy and unhealthy reasons for seeking rhino/septoplasty. He/her has to have a “sixth sense” about motivation, because a substantial number of poor results are based on emotional dissatisfaction rather than a technical failure.

Obviously, every patient seeking RSP cannot be referred for a psychiatric evaluation, nor it is necessary. There are no objective criteria for this “gray zone”. The criteria are subjective and totally different for patient and physician. In the eyes of the low, the responsibility in selecting the patients rests on the surgeon’s shoulders [4, 5].

The aim of our study is to provide an adequate psychological model of person in the patient’s selections during septorhinoplasty.

## Material and Methods

In this prospective, non-randomized study, 140 patients with nasal septal deviation (deviation septi nasi), alone or together with other nasal deformities such as: rhinokyphosis, rhinoskoliosis, rhinolordosis, saddle nose, functional tension nose were observed.

The patients who were on operative list for RSP at the clinic for Ear, Nose and Throat, Clinical Center, Skopje were observed in the period of 4 years. In the patients who wanted to fill the psychological questioner their psychological reactions were taken in consideration. Our intention was for the a patient to recognize and make a mature decision about possible nose changes which would be made with operative intervention and which would also change their emotional and psychological way of living. From 140 patients, 108 were filled the questioner.

Our patients were psychologically followed by two standard psychological tests: Patients selection for septorhinoplasty and their psychological abilities (“Self-body image” questioner) and Brief Symptom Inventory (BSI) test which includes: somatization, obsessive-compulsive reactions, interpersonal sensitivity, depression, anxiety, phobic anxiety, paranoid ideas, and those without symptoms were evidenced.

Jacobson and others [6] developed a questionnaire to be filled in by patients undergoing rhino/septoplasty intervention. We adapted the questionnaire taking into consideration the area they live in and their sociological and cultural habits. It includes the following parameters:

- Patient selection for septorhinoplasty and their psychological abilities:

**(“Self-body image” questionnaire)**

1. How difficult is nasal obstruction or nasal deformity? (filled in by surgeon)
2. The extent of breathing difficulty the patient experiences due to nasal obstruction or nasal deformity
3. Patient’s gender
4. Nationality/religion
5. Cultural habits
6. What is the patient’s self-image in relation to the other people?
7. What are patient’s expectations?
8. Did he/she make the decision for operative intervention?
9. Is his/her decision mature enough?
10. Why is the operation important for him/her?
11. Does any other person know about his/her decision for operative intervention (in the family, at work, etc.)?
12. Has the operation been approved by his/her family?
13. Is the planned surgery his/her first aesthetic operation?
14. What is the surgeon’s opinion about this?

**- Brief Symptom Inventory (BSI) test**- this is a standardized psychological testing, which includes following of some psychological symptoms in patients such as: somatization, obsessive-compulsive reactions, interpersonal sensitivity, depression, anxiety, phobic anxiety, paranoid ideas; the patients without symptoms have been recorded as well.

We followed how RSP make influence in the relation of the patients with his/her family, environment where they work and live before and after surgery.

The period of following the symptoms was before surgery, 1 month after and 6 month after surgery. All the findings were statistically recorded in tables and graphics.

Slight depression which can last 2-4 weeks after operative intervention is expected and therefore we didn’t take it into consideration. The patient is faced with a “new” nose: the surgical procedure, immobilization and recuperation after surgery are stressful for the patient. In childhood years, patients experience “silent pain” and sadness due to changes in part of their body, in this case the nose

## Results

One hundred eight (108) patients, candidates for RSP filled in “The Patients selection for septorhinoplasty and their psychological abilities - “Self-body image” questioner. 60 (55.55%) were women and 48 (44.45%) were men. Age was between 15-57 years, average 31.6 ± 5.8. By nationality, 43 (39.8%) were Macedonian, 44 (40.75%) were Albanian, rest were Turkish and the other nationalities. 97.2% were heterosexual and 2.8% were homosexual.

About their self-concerning, the result shows that most of them 69 (63.9%) were middle concerned, dominant of them, 81 (75%), have real expectations from the operation.

Their Answer of the question “Why you are doing this operation”, 50 (46.3%) thought that the intervention will make to look aesthetically better, both the breathing will be better too (Table 1).

**Table 1:** Patients self-concerning for their nasal deformities

Variable	N	%
How big is their self-concern?		
Little concern	15	13.89
Middle concern	69	63.89
Very concern	24	22.22
Their expectations?		
Real	81	75.0
Unreal	19	17.59
Impossible	8	7.41
Why the operation is important for them?		
Aesthetic moment	26	24.07
Breathing disturbances	32	29.63
Both	50	46.3

According to surgeon’s assessment, 69.45% of the candidates for SRP were psychologically fit for this intervention, 10.2% didn’t have psychological capacity, and 20.4% were followed some period before operation (Table 2).

**Table 2:** Surgeon’s assessment for the RSP candidates

Variable	N	%
Psychologically fit for intervention	75	69.45
Has to be followed some period before operation	22	20.37
Didn’t have psychological capacity for operation	11	10.18
Summary	108	100

Table 3 presented distribution of the patients in comparing with their expectations from rhinosurgery: in 43 (39.81%) thought that self-confidence will be back, 11 (10.18%) thought that RSP will have influence of their profession, 13 (12.04%) thought that RSP will change their lives, 13 (12.04%) thought that their social status will be better and 9 (8.33%) thought that after RSP the other people will behave different with them (Table 3).

**Table 3:** Patient’s distribution according to their expectations from RSP

Patients expectations from RSP	N	%
Self-confidence will be back	43	39.81
Will have influence of their profession	11	10.18
Will change their lives	32	29.63
Their social status will be better	13	12.04
After RSP the other people will behave different with them	9	8.33
Summary	108	100

Chi-square = 42.18, df = 4, p < 0.001.

### Post-operative changes analyzed by Brief Symptom Inventory (BSI) test

We observed that the largest number of the patients (8.7%) expected changes in their lives, (7.7%) thought that realized operative intervention will bring back their self-confidence, few of them (8.8%) thought that the environment will react different with them after surgery. These results were statistically significant.

We have been presented the average scores from different questions which are included in the Brief Symptom Inventory (BSI) test, analyzed in tree periods of time: pre-operatively, 1 month post-operatively, 6 month and 1 year after operation (Table 4).

**Table 4:** The Brief Symptom Inventory (BSI) (score from 1-10)

	Pre-operative	1 month	6 month
Self-confidence	6.8	7.6	7.7
Will have influence of their profession	7.1	7.7	7.7
Will change their lives	8.4	8.6	8.7
Their social status will be better	7.2	7.8	7.7
After RSP the other people will behave different with them	8.5	8.2	8.8

Friedman ANOVA Chi Sqr. (N = 5, df = 2) = 7.89, p = 0.0193; Preoperative/1 month Wilcoxon Matched Pairs Z = 2.02, p = 0.043; Preoperative/6 months Wilcoxon Matched Pairs Z = 2.02, p = 0.043; 1 months/6 months p > 0.05.

The statistical analyze showed that results with high significant scores (p = 0.02). The result scores after 6 months and 1 year after surgery has been not statistically significant.

The results after operative intervention has been qualified, with the modalities: excellent, good, poor and bad, not only from the patients opinion but from the opinions of the surgeon, family, friends too.

Dominantly, most of these analyzed groups the results from the test have been estimated as “excellent”, family estimated in the highest percent 77.8%, surgeons estimated the results with the lowest percent 68.5%. Postoperative results are “bad” for 3.7% of the patients, 1.85% for the surgeons, family and friends (Table 5).

**Table 5:** Post-operative results

The influence of the post. operative results					
	Excellent	Good	Poor	Bad	Summary
	N (%)	N (%)	N (%)	N (%)	N (%)
Patient	78 (72.23%)	22 (20.37%)	4 (3.7%)	4 (3.7%)	108 (100%)
Surgeon	74 (68.52%)	28 (25.93%)	4 (3.7%)	2 (1.85%)	108 (100%)
Friends	78 (72.23%)	24 (22.22%)	4 (3.7%)	2 (1.85%)	108 (100%)
Family	84 (77.78%)	20 (18.52%)	2 (1.85%)	2 (1.85%)	108 (100%)

In Table 6 we showed the distribution of the psychological problems which were registries in the women patients after 1 month in 58 (96.7%) and in 46 (76.7%) after 1 year after surgical operation. These results are statistically significant, means that after 1 year after nose operation women had high significant fewer symptoms according to 1 month after operation (Table 6).

**Table 6:** The Brief Symptom Inventory (BSI) - women

Symptoms	After 1 month	After 1 year
With Symptoms. Without Symptoms.	58 (96.67%)	46 (76.67%)
	2 (3.33%)	14 (23.33%)
Summary	60	60

Chi-square = 10.38, p = 0.0013.

We have been evidenced symptoms in women, which came on control examination made 1 month and 1 year after intervention. The Brief Symptom Inventory (BSI) in women group have shown that most of the women presented symptoms of somatisation one month after [23 (23.33%)], and one year after surgery [15 (25%)]. Obsessive-compulsive reactions were second common symptoms, and were presented in 12 (20%) in women one month and in 9 (15%) one year after surgery. Interpersonal sensibility were shown 4 (6.67%) one month and one year after surgery, Depression were shown in 7 (11.67%) one month and 5 (8.33%) one year after surgery, Anxiety were shown 7 (11.67%) one month and 6 (10%) one year after surgery. Phobic anxiety were shown 5 (8.33%) one month and 6 (10%) one year after surgery. Paranoid ideas were shown only in one woman but we were not sure that symptoms were connected or have been trigged by operation (Table 7).

**Table 7:** The Brief Symptom Inventory (BSI) - women

Symptoms	After 1 month	After 1 year
Somatization	23 (38.33%)	15 (25%)
Obsessive-compulsive reactions	12 (20%)	9 (15%)
Interpersonal sensibility	4 (6.67%)	4 (6.67%)
Depression	7 (11.67%)	5 (8.33%)
Anxiety	7 (11.67%)	6 (10%)
Phobic anxiety	5 (8.33%)	6 (10%)
Paranoid ideas	0	1 (2.17%)
Summary	58 (96.67%)	46 (76.67%)
No symptoms	2 (3.33%)	14 (23.33)

## Discussion

Our expectation in this study was to determine the psychological profile of the patients, by using the above mentioned tests, so that the patients can make a mature decision for having an operation, and acknowledge that the changes will have impact not only on the patient’s faces but on their soul as well. Hence, we developed a “patient’s profile” to help us select which patients are “suitable” and which are “unsuitable” candidates for operative intervention [7-9].

This is useful for the surgeon as well in order to make a reasonable decision about the surgical procedure, especially the post-operative period and in differentiating between surgeon’s expectation and expectation of this delicate group of patients. This shows that we sometimes have to include psychologist and psychiatrist opinions too.

With the Brief Symptom Inventory (BSI) standard testing we showed that beside solving breathing problems and unattractiveness of the face due to nose deformity, RSP has also contributed and positively influenced the patient’s relationships with their friends in environment where they live and work, in their social environment and in emotional life too. The psychological reactions are more dominant in patients, who are themselves anxious, insecure, distrustful and with high expectations from themselves.

There are very few studies that show the relation between the psychological reactions and the septo/rhinoplasty operative technique. We have found similarities in our study and the study prepared by Coin MK and Ress TD in 1991 for patients needing RSP in the Medical Center in New York (the device for plastic and reconstructive surgery and the American Association of the Plastic and Reconstructive surgeons) [10]. Cash and Horton discovered that those patients who are depressive by nature, do not want to openly discuss their psychological problems. They very often hide a lot of anamnestically significant things from the doctor and direct their interest towards the aesthetic problem, thus somatising their inner psychological problem [11, 12].

Belgian researchers studied 266 patients over a period of 16 months and found that more than 43 percent of the patients-candidates for RSP have some kind of dysmorphic disorder. The study, published in the August issue of Plastic and Reconstructive Surgery, shows a surprisingly high rate of body dysmorphic disorder among nose-job patients. Previous studies have shown that about 10 percent of patients seeking plastic surgery suffer from the condition [13].

Very often, by subconsciously blaming the surgeon that he/she didn’t do their nose “well” they hide serious dysmorphobic emotional disturbances.

We showed that according to Patients selection for septorhinoplasty and their psychological abilities (“Self-body image” questionnaire) and Brief Symptom Inventory (BSI) test, the patients made a reasonable decision about their aesthetic intervention, which was useful for the surgeon too, and it helped them choose an adequate operative technique and especially helped them in postoperative period. Consequently, those tests determine the realistic surgeon’s and patient’s expectations [14, 15].

In conclusion, the experience, skill and adequate operative technique are very important for the surgical procedure and the success of the operation. Nevertheless, the good communication between the surgeon and the patient is equally important, because it affects the psychological condition of the patient in the post-operative period.

This “vital connection” can be shattered by the surgeon’s arrogance, hostility, or indifference (real or imagined), but especially by the patient’s feeling that “the surgeon didn’t care”. The second important thing for the surgeon is to learn to avoid the patient who is going to feel this way no matter what is done.

For this reason, we sometimes need to work as a team when selecting patients-candidates for septorhinoplasty, so as to avoid the existing problems like dysmorphobia which can have pathological consequences [16, 17].
